# A High-Efficiency Uneven Cluster Deployment Algorithm Based on Network Layered for Event Coverage in UWSNs

**DOI:** 10.3390/s16122103

**Published:** 2016-12-12

**Authors:** Shanen Yu, Shuai Liu, Peng Jiang

**Affiliations:** College of Automation, Hangzhou Dianzi University, Hangzhou 310018, China; shanen_yu@hdu.edu.cn (S.Y.); liushuaihdu@163.com (S.L.)

**Keywords:** underwater wireless sensor networks (UWSNs), event coverage, layered, uneven cluster, recovery strategy

## Abstract

Most existing deployment algorithms for event coverage in underwater wireless sensor networks (UWSNs) usually do not consider that network communication has non-uniform characteristics on three-dimensional underwater environments. Such deployment algorithms ignore that the nodes are distributed at different depths and have different probabilities for data acquisition, thereby leading to imbalances in the overall network energy consumption, decreasing the network performance, and resulting in poor and unreliable late network operation. Therefore, in this study, we proposed an uneven cluster deployment algorithm based network layered for event coverage. First, according to the energy consumption requirement of the communication load at different depths of the underwater network, we obtained the expected value of deployment nodes and the distribution density of each layer network after theoretical analysis and deduction. Afterward, the network is divided into multilayers based on uneven clusters, and the heterogeneous communication radius of nodes can improve the network connectivity rate. The recovery strategy is used to balance the energy consumption of nodes in the cluster and can efficiently reconstruct the network topology, which ensures that the network has a high network coverage and connectivity rate in a long period of data acquisition. Simulation results show that the proposed algorithm improves network reliability and prolongs network lifetime by significantly reducing the blind movement of overall network nodes while maintaining a high network coverage and connectivity rate.

## 1. Introduction

**Figure 1:**
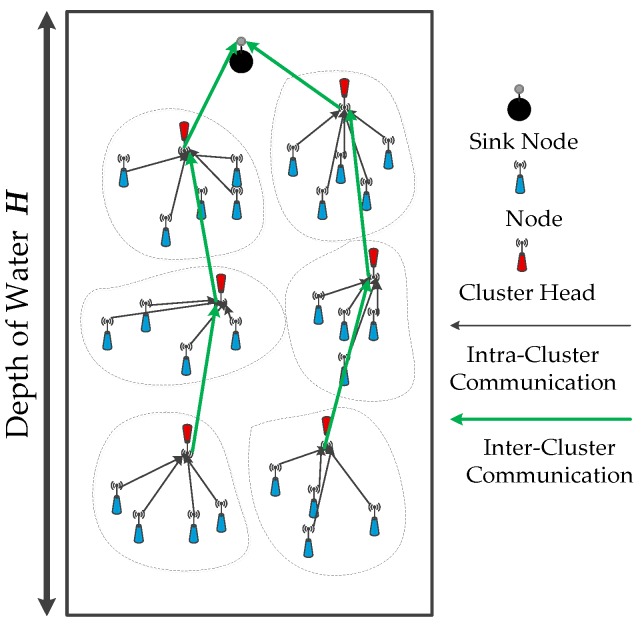
Transmission characteristic based on clustering in UWSNs.

The applications of underwater wireless sensor networks (UWSNs) mostly include oceanography data collection, intrusion detection applications, disaster prediction, and mineral exploitation [1,2,3,4], which involve nodes that are capable of underwater data collection and communication processing through underwater acoustic signals [5,6,7]. The underwater acoustic signal communication involves a low propagation speed of sound at approximately 1500 m/s, with slow attenuation in underwater environments. Compared with node deployment problems of terrestrial WSNs [8,9], the node deployment of UWSNs has many different characteristics. First, node deployment in dynamic three-dimensional (3D) water environments [10], with fundamental properties of 3D networks, still have content, such as mobile ad hoc networks and aeronautical ad hoc networks [11,12]. Second, the acoustic signal is usually selected as the communication medium, as the optical or electromagnetic wave signal attenuates rapidly in underwater environments. Third, limited node energy is an important factor for network operation; it is expensive and impractical to recharge batteries in harsh aqueous environments [13,14]. Therefore, in this study, energy consumption efficiency is a critical target for UWSNs node deployment. Usually, the relay and transmission direction in UWSNs is *down-to-up* [15,16]. As shown in Figure 1, the individual nodes in different depths of water will bear different opportunities to send and receive a message. If the node is closer to the water surface, then its energy consumption is faster and eventually leads to premature death. Local network coverage holes and disconnection of communication links during network operation reduces the performance of coverage and connectivity until the entire monitoring network eventually fails. However, many researchers have begun to investigate uneven node deployment strategy based on event coverage.

Xia et al. [15] first proposed a fish swarm-inspired node deployment algorithm, which is typical deployment algorithm based event coverage. Subsequently, based on [17], Xia et al. proposed particle swarm-inspired node deployment (PSND) in [18]. These kinds of algorithms optimize network performances via simulating the behavior of fish or particle swarms and introducing the model of congestion degree control. These two proposed algorithms can drive nodes to cover the events and match event distribution density in monitoring the water environment. The author only considers the coverage rate of events, and obtaining the high network connectivity rate is difficult. In addition, these algorithms drive a number of redundant node moves, and nodes may move without a clear purpose during the deployment processing. Given the limited energy of nodes and the difficulty of recharging them, these defects may cause premature deaths of nodes, thus shortening the network lifetime.

Jiang et al. [19] proposed node non-uniform deployment based on a clustering algorithm. First, the deployment algorithm is based on clustering. Then, a heterogeneous process is run for node communication. These steps ensure full network connectivity and define the aggregate contribution degree. Then, the algorithm substitutes the node with the smallest aggregate contribution degree for the node of premature death. The algorithm effectively improves the network performance and prolongs network lifetime. However, the algorithm assumes that all of the nodes possess the ability of free movement. Notably, this assumption significantly increases network costs and may not be practical.

Bharamagoudra et al. [20] proposed a deployment scheme to enhance coverage and connectivity (DSECC). The deployment scheme obtained a mathematical model of node deployment based on underwater acoustic communication properties. They used multiple surface gateways and autonomous underwater vehicles (AUVs) to enhancing network coverage and connectivity. Then, they planned the network routing path of AUVs to collect real-time information and repair premature dead nodes. However, the DSECC algorithm requires global node coordinates and does not consider the influence of water flow.

The aforementioned algorithms fail to consider the characteristics of data relay and the transmission direction of UWSNs (*down-to-up*). The network will bear more relay and transmission and consume more energy if the node close to the water surface sinks, thus leading to different distributions of energy consumption at different underwater depths and to imbalances between the energy consumption of each layer network. The network communication links near the water surface disconnect easily during period of network operation, thus influencing data acquisition and decreasing network lifetime.

Regarding aforementioned problems of deployment algorithms based the event coverage, in this study, we proposed an uneven cluster deployment algorithm based network layered (UCBNL) for event coverage in UWSNs. According to the monitoring of water environments under the random distribution of isolated event information, node deployment should guarantee real-time collection of event information and ensure that the final data transmission to the surface sink nodes set. In other words, we should ensure high network coverage rate to improve network connectivity rate while effectively conserving node energy and balancing global network energy consumption. Based on the characteristics of UWSNs data acquisition (*down-to-up*) in underwater environments, we proposed UCBNL algorithm comprising two parts. First, we use hierarchical static deployment based on probability theory and mathematical statistics analysis, where each layer node deployment expectation is calculated to meet the network performance quality of UWSNs and parameters of deployment density are adjusted accordingly. Second, the uneven deployment method was proposed for the communication load distribution and connectivity or coverage deployment requirements of each node. This method narrows the move range of nodes and increases the size of the node density and cluster number of each layer if they are closer to the sink node of the water surface. Thus, we obtain the capabilities of a temporary fix node, conserve the movement energy and deployment time, and balance the energy consumption of nodes in each layer. Simulation results show that the UCBNL algorithm can maintain high network coverage and connectivity rates for a long period of time, reduce network energy consumption, and decrease the slope of network recession, thereby prolonging network lifetime. Compared with existing deployment algorithms for event coverage in UWSNs, our proposed algorithm contributes the following:

(1) The prior node distribution density of a layered network is derived, thus reducing the movement distance of node and balancing network energy consumption.
(2) The adjustable communication radius model ensures that the network is fully connected at the initial process of network deployment. The network connectivity rate can be improved adaptively in the subsequent data acquisition.
(3) The recovery strategy of clusters utilizes the node with maximum comprehensive advantage to help recover the death node, thus prolonging network lifetime and improving the efficiency of data acquisition.

The remainder of this paper is organized as follows: in Section 2, related studies regarding node deployment problem are described. In Section 3, models of UWSNs and related definitions are described. In Section 4, the problem is analyzed and the details of the UCBNL algorithm are provided. In Section 5, the description of algorithm simulation and a detailed analysis of the simulation results are discussed. Finally, in Section 6, conclusions and future works are discussed.

## 2. Related Work

Current research of UWSNs has focused on acoustic communication, network protocol, routing algorithms, and node localization and tracking [21,22,23]. However, the strategy of node deployment directly correlates to network operation performances and thus has a strong influence on designs of network protocol and topology control subsequently. Therefore, in this study, we will investigate node deployment. For the different strategies of node deployment algorithms, Han et al. [24] summarize the node deployment algorithms for UWSNs based on the movement ability of the nodes. They created three categories, namely, static deployment, self-adjustment deployment, and movement-assisted deployment.

First, the static deployment algorithm [25,26,27] assumes that all nodes that cannot move require an artificial deploy node to optimize position by advanced calculation. This algorithm can usually achieve high network performance. However, this algorithm also adds many difficulties for artificial deployment to poor monitoring aquatic environments, thus increasing high deployment costs. Second, in self-adjustment deployment [28,29,30], the node can only move in a vertical direction by adjusting the length of the anchor rope. This kind of algorithm increases the network coverage area or decreases the coverage overlap by adjusting the depth of the node. Usually, self-adjustment deployment is an economical strategy, but cannot achieve good redeployment performance when the network operation period is long or several nodes are dead. Finally, movement-assisted deployment is usually equipped with multiple sink nodes and AUVs, thus achieving high network performance. Moreover, movement-assisted deployment has an important role in network redeployment and can achieve the repair of network coverage holes and the reconnection of disconnected communication links. Therefore, we will use the movement-assisted deployment strategy to achieve high network performance of event coverage under long periods.

## 3. Models and Related Definitions

This section provides some related models of node deployment and the gives related definitions.

### 3.1. Models

#### 3.1.1. UWSNs Models

**Figure 2:**
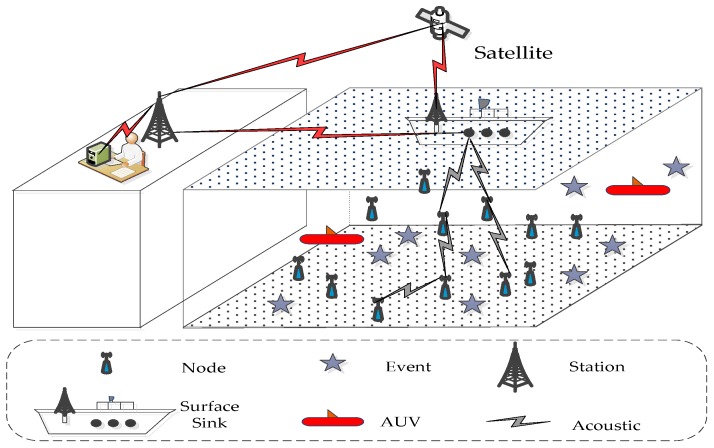
3D UWSNs architecture by using the layered strategy.

In this study, combined with the characteristics of 3D UWSNs, we propose that the design scheme comprises nodes suspended in the underwater area and sink nodes set floating on the surface and multiple AUVs. As shown in Figure 2, the final topology structure of the UWSNs is presented in a 3D rectangular monitoring water area. A number of sink nodes are uniformly deployed along the water surface center line, and the network gradually forms an approximate hierarchical state. In this study, we also have the following preliminaries:

(1) Each node can perceive the self-depth information; thus, we can determine its layer number.
(2) Each node has a unique ID. The nodes use unified underwater acoustic signals to communicate and acquire information. Sink nodes transmit the information to the base station by radio frequency signals. If acquisition information is sent to any sink node set on the water surface, then data acquisition is successful.
(3) All nodes have the same initial energy *E_init_* and sensing radius *Rs* (using the Boolean sensing model). The nodes are equipped with a transmission power control module [7] that can adjust the communication radius *R_C_* within a 5 m accuracy range that does not exceed a fixed threshold *R_Cmax_*. Furthermore, the energy of the sink node is sufficient, and its energy consumption is negligible.

#### 3.1.2. Node Perception Model

We adopt Boolean perception to describe node sensing. Assuming the coordinate of node *s_i_* is $\left(x_{i},y_{i},z_{i}\right)$ and the Euclidean distance between *s_i_* and point *p*(*x,y,z*) is $d(s_{i},p)=\sqrt{{\left(x-x_{i}\right)}^{2}+{\left(y-y_{i}\right)}^{2}+{\left(z-z_{i}\right)}^{2}}$, then the probability that point *p* is perceived by node *s_i_* can be presented, as shown in Equation (1), as follows:

(1) $$c_{p}(s_{i})=\left\{ \begin{array}{ll} 1, & d(s_{i},p)<\;Rs\\ 0, & d(s_{i},p)\geq Rs \end{array}\right.$$

where *Rs* denotes the sensing radius of node *s_i_*.

#### 3.1.3. Node Energy Consumption Model

The energy consumption of node receiving information is smaller than data transmission and node movement [31]. Therefore, we only consider data transmission and node movement the primary components of energy consumption. In this study, the energy consumption model of node is mentioned in [32], which can be calculated through using Equation (2), as follows:

(2) $$E_{c}(d,f)=P_{0}T_{p}d^{k}10^{\alpha (f)d/10}$$

where *P*_0_ denotes the power threshold for a node to receive the information package, *T_p_* denotes the transmission delay of the information package, *d* denotes the transmitting distance of the information package, and *k* denotes the energy spreading factor. The absorption coefficient *α*(*f*) can be calculated by using Equation (3), as follows:

(3) $$\alpha (f)=0.11\frac{f^{2}}{1+f^{2}}+44\frac{f^{2}}{4100+f^{2}}+2.75\times 10^{-4}f^{2}+0.003$$

where *f* is the frequency of the carrier acoustic signal in Hz and *α*(*f*) is in dB/m. In addition, the movement energy consumption *M_e_* of the node can be expressed as the product of the movement distance *m_d_* and the energy consumption of per movement distance *m_u_*. The relationship is described through the Equation (4), as follows:

(4) $$M_{e}=m_{d}\times m_{u}$$

#### 3.1.4. Adjustable Communication Radius Model

We assumed that the maximum communication radius of the node is *R_C_* and its initial value is *R_Cinit_*. The adjustable level of the communication radius is defined as *θ* and is initialized to be 1. Therefore, we can obtain the relationship between *R_C_* and *θ* by using Equation (5), as follows:

(5) $$R_{C}=R_{Cinit}+\left(1+\theta \right)B_{u}$$

#### 3.1.5. Node Mobility Model under the Influence of Water Flow

In this study, we adopt the water flow model in [33] to describe the mobility state of nodes. After network deployment is completed, the sink node with sensor nodes moves horizontally. Then, the speed of a node in the *x*- and *y*-axis directions is calculated by Equation (6), as follows:

(6) $$\left\{ \begin{array}{l} V_{x}=k_{1}\lambda v\sin(k_{2}x)\cos(k_{3}y)+k_{1}\lambda \cos(2k_{1}t)+k_{4}\\ V_{y}=-\lambda v\cos(k_{2}x)\sin(k_{3}y)+k_{5} \end{array}\right.$$

where *k*_1_, *k*_2_, *k*_3_, and *v* are the variables related to the water environment and *k*_4_ and *k*_5_ are random variables. These variables are set as follows: $k_{1},k_{2}∼N(\pi ,0.1\pi )$, $k_{3}∼N(2\pi ,0.2\pi )$, $k_{4},k_{5}∼N(1,0.1)$, λ∼N(3,0.3), and v∼N(0,0.1). Then, the location of the node is updated by using Equation (7), as follows:

(7) loc(i)=loc(i−1)+T*v(i)

where *loc*(*i*) denotes the node location at time *i*, *loc*(*i* − 1) denotes the node location at time (*i* − 1), and *T* denotes the update period of the node location and is set to 60 s.

### 3.2. Related Definitions

#### 3.2.1. Network Connectivity Rate

Network connectivity rate *C_n_* is defined as the ratio of *n_c_* to *n*, where *n_c_* denotes the number of nodes that can communicate with the sink node through single-hop or multi-hop communication. *n* is the total number of nodes in the monitored underwater environment space. Therefore, *C_n_* can be calculated by using Equation (8), as follows:

(8) $$C_{n}=\frac{n_{c}}{n}$$

If *C_n_* is equal to 1, then the network achieves full network connectivity, that is, all nodes can communicate with sink nodes through single-hop or multi-hop communication.

#### 3.2.2. Network Coverage Rate

In this study, the object of monitoring is isolated. Discrete events occur underwater and their uniform distribution is random. Network coverage rate *C_c_* of the UWSNs is calculated by using Equation (9), as follows:

(9) $$C_{c}=\frac{E_{c}}{E_{t}}$$

where *E_c_* is the number of events covered by all nodes and *E_t_* is the total number of events distributed in the monitored underwater environment space.

#### 3.2.3. Network Lifetime

Network lifetime is an important criterion to evaluate the energy efficiency of algorithms [34,35]. In this study, network lifetime is defined as the number of data acquisitions performed when coverage rate *C_c_* meets the conditions ($C_{th}\leq C_{c}\leq 1$) and *C_th_* is a presetting threshold of network coverage rate.

#### 3.2.4. Network Reliability

As a crucial indicator for evaluating the network quality of service [36], network reliability is the probability that the network can maintain full network connectivity at a high level. This study mainly investigates UWSNs deployment issues. Thus, the network should still maintain a high network connectivity rate. This optimization goal is crucial in our algorithm, as several nodes begin to die because of persistent network operation. Therefore, in this study, network reliability can be described as the average node degree and is calculated by using Equation (10), as follows:

(10) $$D_{a}=\frac{\sum_{i=1}^{n}N_{i}(nei)}{n}$$

where *D_a_* denotes the average node degree and *N_i_*(*nei*) denotes the neighbor node number of node *s_i_*.

#### 3.2.5. Movement Coverage Gain

The movement coverage gain is defined as the growth rate of coverage at each node movement by the help of AUVs. Then, the movement coverage gain *C_g_*(*i*) of node *i* can be calculated by using Equation (11), as follows:

(11) $$C_{g}(i)=\frac{(C_{B}-C_{A})*V}{4/3\pi Rs^{3}}$$

where *C_B_* denotes the network coverage rate before the node moves, *C_A_* denotes the network coverage rate after the node moves, and *V* denotes the area of the network monitoring space.

## 4. Problem Analysis and Algorithm Description

This section describes deployment problem based event coverage, and resolves corresponding defects. Further proposed UCBNL by give some detail algorithm descriptions.

### 4.1. Problem Analysis

In theory, we can obtain full network coverage and connectivity if the monitoring space deployed a sufficient number of nodes, but the costs of network deployment nodes must be considered a factor for network monitoring tasks. Obviously, arbitrarily deploying an excessive number of nodes is impossible. So, we must set a reference value of the number of nodes to be deployed. Recently, research on uneven deployment in UWSNs has mainly focused on the location of nodes based on optimized network coverage, connectivity rate, and lifetime. The algorithm drives the nodes to the right location to maximize network coverage and connectivity rate. Usually, the predecessors do not consider the characteristics of data acquisition from the bottom to the top in underwater environments. The nodes closer to the underwater surface will undergo more data transmission and relays. Therefore, different depths of the clusters will impair the energy consumption balance of the overall network. With the network operation, nodes close to the surface of the water and the communication link are easily disconnected, affect data acquisition, and reduce network lifetime. Existing deployment algorithms based on event coverage for UWSNs, such as DSECC and PSND, usually only consider improving network coverage or connectivity but ignore the effective utilization of overall network energy. The movement efficiency of these algorithms is unsatisfactory because they do not set a reference value for node deployment and because the large nodes movement energy consumption occupies the energy consumption required for communication and data acquisition. Therefore, defects may cause communication link disconnection and premature deaths of clusters because of node energy exhaustion, which shortens the overall network lifetime. In this study, the UCBNL is proposed for event coverage. We introduce a layered mechanism to maximize network connectivity and coverage rate under optimal conditions of node mobile energy consumption. We analyze the communication load distribution in each layer underwater network and the deployment requirements of coverage and connectivity. The node distribution density $\rho_{i}$ of each layer is obtained, and we obtain the node deployment expectation *E_e_* of each layer.

### 4.2. Algorithm Description

The UCBNL consists of two phases, namely, the priori analysis and the uneven cluster deployment.

#### 4.2.1. Interlayer Distance *d* and Deployment Expectation $E_{e}(i)$

Given the diversity, dynamics, and uncertainty in the data acquisition process of network operation for harsh aqueous environments, accurate prediction of energy utilization is impossible. Therefore, we provide the calculation model of energy consumption along with a reasonable solution. In the phase of network initialization, nodes moving to a certain range because of water flow constantly change communication distance and redundant coverage. Inspired by the hierarchical deployment strategy, we first build a coverage and connectivity threshold of the bottom layer. Then, we set a bottom layer node initial deployment standard value $\left\|D_{\Delta }\right\|$. For convenience, we can adjust the standard value $\left\|D_{\Delta }\right\|$ based on the density of event information and dynamically adapt the changes of the underwater monitoring environment to satisfy the requirements of coverage deployment. In the process of network layering, the algorithm will produce interlayer coverage redundancy if the interlayer distance is insufficiently small. The algorithm cannot reflect the concept of the hierarchical strategy that eliminates the “hot spots” phenomenon and cannot balance node transmission and relay node energy consumption if the interlayer distance is excessively large. After comprehensive consideration in this study, we should select a suitable and fixed interlayer distance *d*.

**Figure 3:**
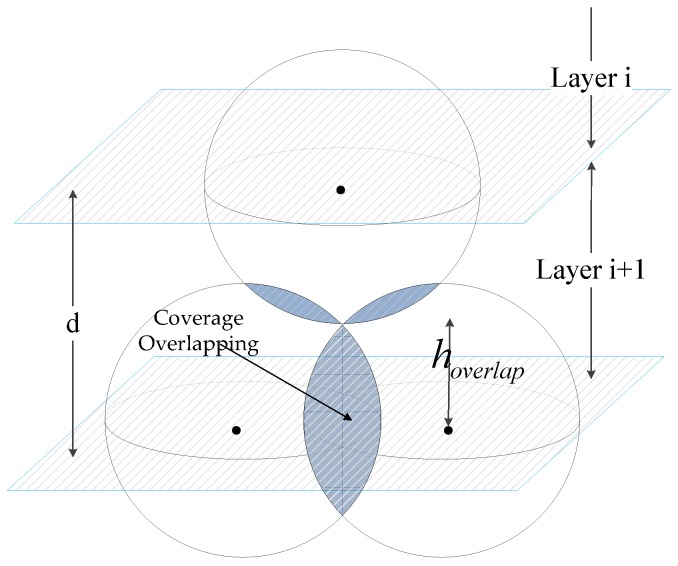
Calculation of interlayer distance in layered network.

In this study, we assume that the volume of the deployment water area is *L* × *W* × *H* and perform hierarchical processing in *H* water depth. The sink node set is deployed on the surface of monitoring water. The water surface layer is assumed to be equal to level 0. All nodes are deployed in the sink nodes set below and form different network layers. *H_i_* denotes the node depth in layer i (i=1,2,...,l) and l=⌊H/d⌋. When *i* = *l*, *Hl* denotes the node depth in layer *l* and sink nodes set far from the surface of the water. As shown in Figure 3, we can analyze the overlapping node coverage relationship of two adjacent layers. Then, we obtain the network interlayer distance comprising two parts, namely, the *i*-th layer nodes coverage overlap distance *h_overlap_* and the node sensing radius *Rs*. The interlayer distance *d* can be calculated by using Equation (12), as follows:

(12) $$d=h_{overlap}+Rs$$

Given that value selection of *h_overlap_* is important for valuing interlayer distance *d*, *h_overlap_* is decided by expectation distance *E_D_* between any two nodes in layer *i*. The bottom layer network can meet the optimal deployment (minimum coverage overlap), with a minimum node deployment number to satisfy the coverage requirements in this layer network. The expectation distance *E_D_* can be obtained by the equation, *E_D_* = *Rs*, to reflect the concept of hierarchical equilibrium. Then, we adopt the formula $h_{overlap}=\sqrt{Rs^{2}-{\left(E_{D}/2\right)}^{2}}$. We eventually obtain interlayer distance *d* calculated by using Equation (13), as follows:

(13) $$d=\sqrt{Rs^{2}-{\left(E_{D}/2\right)}^{2}}+Rs=\left(1+\sqrt{3}/2\right)Rs$$

We let *n* denotes the total number of nodes in the network and D=L×W×H the volume of the network field (i.e., the volume of *i*-th layer is *D_i_*). We obtain the following Equation (14):

(14) n=ρD

The expected number *n_i_* of nodes in *D_i_* is $n_{i}=\rho_{i}D_{i}$. For uniform distribution of event sources, the expected number $\left\|T_{i}\right\|$ of event sources in *D_i_* is shown in Equation (15), as follows:

(15) $$\left\|T_{i}\right\|=\left\|T\right\|\frac{D_{i}}{D}=\frac{\left\|T\right\|}{l}$$

In any layer network *D_j_* (*j > i*), source-to-sink paths associated with event sources have the sink node set as their destination. Underwater sensors collectively participate in all of these paths as message forwarders. The expected number of such paths per node in $D_{j}$ is described in Equation (16), as follows:

(16) $$m_{f}(i)=\frac{1}{n_{i}}\sum_{i<j\leq l}\left\|T_{j}\right\|=\frac{\left(l-i\right)\times \left\|T_{i}\right\|}{n_{i}}$$

In *D_i_*, the expected number *m_o_*(*i*) of paths originated per node is denoted in Equation (17), as follows:

(17) $$m_{o}(i)=\frac{\left\|T_{i}\right\|}{n_{i}}$$

Thus, we can obtain the expected energy consumption *E*(*i*) of each node in *D_i_* as denoted in Equation (18), as follows:

(18) $$E(i)=\left(m_{f}(i)+m_{o}(i)\right)E_{c}(\omega_{i})$$

On the basis of Equations (15)–(18), we obtain the following equation:

(19) $$E(i)=\left[\frac{1}{n_{i}}\left(\left\|T_{i}\right\|+\sum_{i<j\leq l}\left\|T_{j}\right\|\right)\right]E_{c}(Rc,f)=\frac{\left\|T\right\|H_{i}}{Hn_{i}}\left(\left\lfloor \frac{H}{d}\right\rfloor +1-i\right)E_{c}(Rc,f)$$

Balancing energy consumption by properly applying different node densities is a key problem. We denote node density in the *i*-th layer *D_i_* by $\rho_{i}$. The set should contain more nodes for sharing message relays than a relatively distant one in different depths underwater ($i.e.,\;\rho_{l}<\rho_{l-1}<\cdots \rho_{2}<\rho_{1}$) to balance the energy consumption of nodes close to the sink node. Thus, we only need to determine the function relationship between $\rho_{i}$ and $\rho_{l}$ such that E(i)=E(l) for 1≤i≤l. Applying a simple derivation and calculation from the previous equation, we obtain a ratio function λ of $\rho_{i}$ and $\rho_{l}$ in Equation (20), as follows:

(20) $$\lambda =\frac{\rho_{l}}{\rho_{i}}=\frac{1}{l+1-i}.$$

We only need to satisfy the deployment requirement of monitoring underwater environments (events information distribution) to set up a standard value $\left\|D_{l}\right\|$ of initial deployment underwater. At the same time, we can dynamically adjust the deployment of the standard value $\left\|D_{l}\right\|$ by adding tuning parameter λ. Thus, we can obtain the expected number $E_{e}(i)$ of nodes in the *i*-th layer, as shown in Equation (21):

(21) $$E_{e}(i)=\lambda \left\|D_{l}\right\|$$

#### 4.2.2. Uneven Clustering Deployment Based on Event Distribution

**Figure 4:**
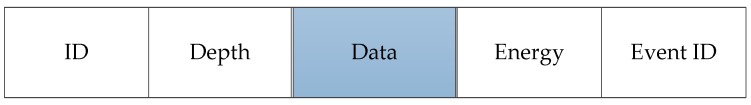
Data structure of the node.

First, there data structure of the node is described, as shown in Figure 4. Then, the two related definitions are subsequently described.

**Definition** **1.** *Relative residual energy. The current residual energy of any node i is denoted by* $\xi_{r}$ *and the average residual energy of all nodes in their layer is denoted by* ξ*. The minimum residual energy of all nodes in this layer is denoted by* $\xi_{m}$ *and this value can be obtained through sink node periodic feedback information to the nodes of the overall network. Then, the relative residual energy* $\xi_{\Delta }$ *of the nodes in their layer is defined by* $\xi_{\Delta }=\left(\xi_{r}-\xi_{m}\right)/\left(\xi -\xi_{m}\right)$*.*

**Definition** **2.** *Relative depth. For any node j in layer i in the depth range* [i×d,(i+1)×d]*, the distance between the bottom of layer i and the node j is denoted by d_r_(j). Then, the relative depth of the node j is defined as* $d_{\Delta }(j)=\left(d_{r}(j)/d\right)$*.*

**Figure 5:**
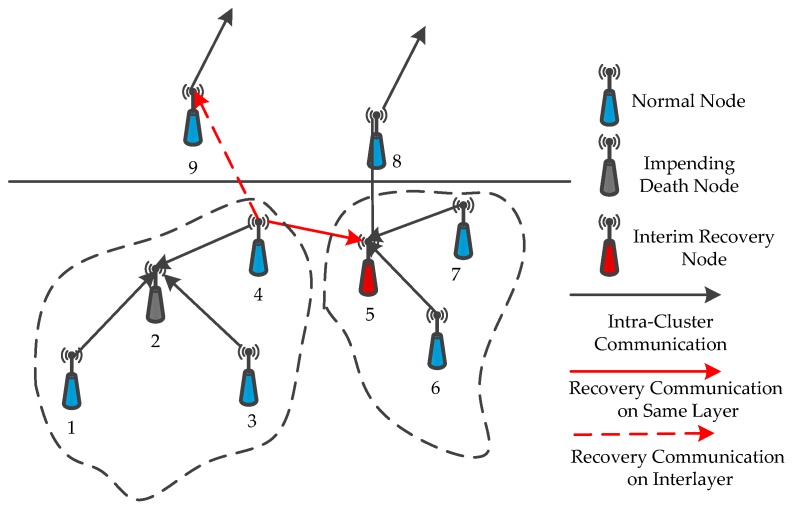
Recovery strategy of clusters.

**Figure 6:**
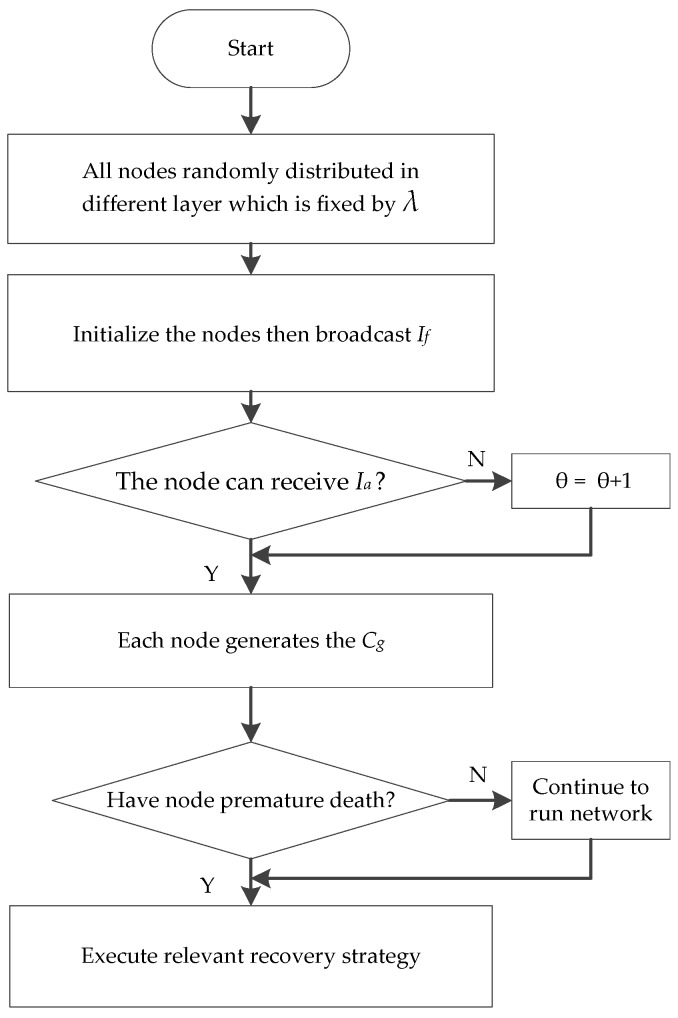
Algorithm of uneven cluster flowchart.

Given that event location information is unknown in UWSNs, the network is layered and all nodes are deployed by random uniform deployment. Combined with the location of event information and interlayer distance to deploy nodes and define the work mechanism of nodes effectively, we propose a non-uniform clustering deployment algorithm or strategy based on event location information. Generally, adopting the clustering algorithm will let cluster head nodes consume more energy on data forwarding and relaying. Thus, we must consider the influence of more energy consumption of cluster head nodes throughout network operation. The implementation procedures of the algorithm are as follows:

(1) In the monitored underwater environment whose volume is *L* × *W* × *H*, multiple sink nodes create a uniform linear layout on the water surface center line. Section 4.2.1 shows that we can obtain a total deployment expected number $\left\|D_{i}\right\|$ of nodes for network layer *i*. Then, all of the nodes adjust their depth randomly within the interlayer range, and the number of events distributed in the space is *e_t_*.
(2) The communication levels *θ* of all of the nodes in layer *l* are initialized to be 1. All communication node radii are *Rc*. All nodes in layer *l* broadcast the neighbor finding messages *I_f_* and attempt to receive that message from other nodes in this layer network. If node *i* receives the neighbor finding message *I_f_* from node *j* in the range of broadcast radius, then the same time node *i* must return a response message *I_a_* to node *j* with the same communication radius. If node *i* cannot receive any neighbor finding message from all other nodes in its layer *l*, then node *i* is denoted as a state of neighbor barely. Thus, the node gradually increases the communication level *θ* to broadcast neighbor finding message *I_f_*. The aforementioned process continues the iteration until all nodes in this layer network can receive response messages *I_a_*. The neighbor node is defined by $neig_{j}^{l}$ if node *j* can receive response message *I_a_* from the network of layer *l*. If the response message *I_a_* comes from a layer network above, then the neighbor node is defined by $neig_{j}^{l-1}$. We record the information of all of the nodes of each layer network by using the same principle until the overall network nodes can receive response message *I_a_*. However, the nodes in the first layer network have no upper neighbor nodes; thus, its upper neighbor node information will not be labeled.
(3) All nodes perceive their surrounding event and broadcast and receive cluster generation information *C_g_* (contains the number of perceived events and the neighbor node information). Given that cluster head nodes have more energy consumption that leads to premature death, they will be a prior consideration. Node *i* selects the node that can perceive most events from its neighbor nodes (including itself) to be its cluster head to maintain the effective network coverage rate. If multiple neighbor nodes can perceive the same maximum number of events, then node *i* selects the node away from its nearest neighbor as its cluster head node.
(4) After completing the previous steps, each cluster head node will collect event information and transmit data to sink node sets by the single-hop or multi-hop method. The cluster head node chooses the node (including the cluster head node and intra-cluster node) with maximum forwarding probability *p_j_* as its next-hop and the next-hop node in other clusters in the upper layer network. We can determine the forwarding probability *p_j_* by the following formula: *p_j_* = Δ/*c*, where we define the comprehensive advantage as $\Delta =a\xi_{\Delta }+bd_{\Delta }$. *a* and *b* is a regulatory factor, and *a* + *b* = 1, *c* is the number of neighbor nodes that the cluster head nodes can detect in their upper layer network. The intra-cluster node selects its cluster head node to form the next-hop path.
(5) When the network undergoes many rounds (the number of data acquisition) or is affected by underwater current, volcanic eruption, or harbor and shore activities, the cluster head node may die prematurely and the network topology will change. Once the data transmission chain shows the phenomenon of “interlay disconnect”, then the data transmission will fail between adjacent layers. We need to select several proper relay nodes to substitute for the forwarding nodes out of service or to reselect the new cluster head node. The specific recovery strategy is as follows: When the data transmission fails in interlay network communication, the cluster head nodes select the cluster member node with a more relative residual energy $\xi_{\Delta }$ as the temporary cluster head node. If the data transmission chain restores data acquisition in the interlay network, then the temporary cluster head node *j* is directly replaced as the real cluster head node; otherwise, the temporary cluster head node *j* detects neighbor clusters in the same layer and selects the nearest node in neighbor clusters away from node *j* to forward messages (as shown in Figure 5, node 2 is a cluster head of nodes 1, 3, and 4 when the energy of cluster head node 2 is almost exhausted. Node 2 initially attempts to select node 4 as the temporary cluster head with more relative residual energy $\xi_{\Delta }$. If the data transmission chain is still unable to connect, then the cluster head node 2 selects node 5 as its next-hop node at the neighbor cluster in the same layer network). If the two strategies still cannot achieve the data transmission, then we can conclude that the cluster has reached the threshold of death. We subsequently select other nodes to replace the original node that almost reached the threshold of death. Then, we recover the cluster. For the cluster that reaches the threshold of death, the cluster head node *C(j)* of node *j* determines the largest comprehensive advantage Δ of node *i* in its cluster. If node *i* can recover the transmission chain by itself to substitute for node *j*, then determining the node *i* to move in a straight line to substitute for node *j.* If the transmission link is still disconnected, then the cluster head node searches all neighbor cluster nodes within the same layer and generates a report on all cluster generation information *C_g_* of the nodes, including the number of events covered and depth information. Each neighbor cluster head node sends help message *H_m_* (which may be several) to node *j* and selects the nearest node *h* to substitute for node *i*. If cluster head node *C*(*j*) cannot receive any help message, then the process of recovering fails. Furthermore, the overall algorithm description of uneven cluster deployment based on event distribution is shown in Figure 6.

## 5. Simulation Evaluation

This section gives related parameter settings and evaluation metrics, and then these metrics go on with a detailed analysis of the simulation results.

### 5.1. Parameter Settings and Evaluation Metrics

We will compare UCBNL with PSND and the DSECC on five metrics, namely, network energy consumption, network coverage rate, network connectivity rate, network lifetime, and network reliability. In addition, the recovery efficiency can be expressed by movement coverage gain in Section 3.2.5. To verify the effectiveness of the UCBNL algorithm, MATLAB is used to simulate and analyze experiment results, and the volume of monitored water space is 200 m × 200 m × 200 m. The metrics of the simulation results are the mean of 50 experiments to eliminate experimental error. Other default parameters are set in Table 1 [19].

### 5.2. Comparison and Analysis of the Simulation Results

**Figure 7:**
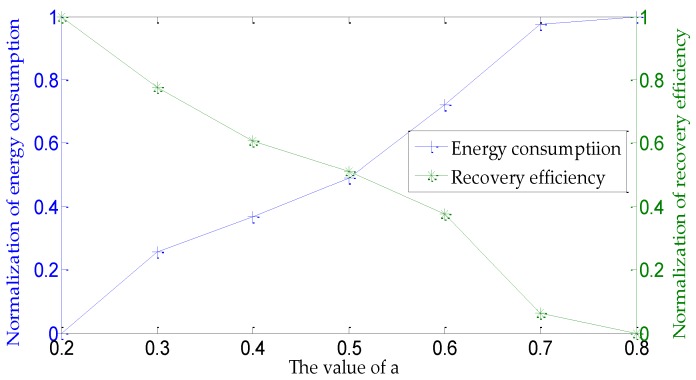
Influence of different values of *a* on network performances.

Figure 7 shows the influences of the different values of *a* on network energy consumption and recovery efficiency when executing the UCBNL. All of the other parameters have default settings. Figure 7 shows that, when *a* is 0.5, the comprehensive results of network energy consumption and recovery efficiency are more balanced. Therefore, in subsequent simulation experiments, the default setting of *a* is 0.5.

#### 5.2.1. Network Energy Consumption

**Figure 8:**
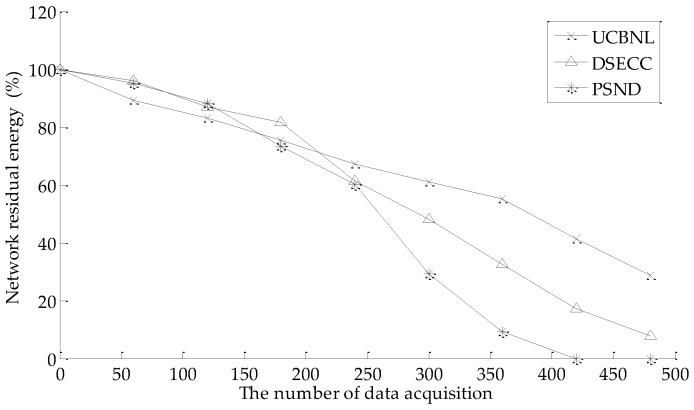
Comparison of energy efficiency during data acquisition.

Figure 8 shows that the comparison of the percentage of network residual energy varies with the network operation number (the number of data acquisition) on the UCBNL, PSND, and the DSECC, where the number of events is 400 and the number of nodes is 60 in the simulation scenario. As shown in Figure 8, the network residue energy is similar among UCBNL, PSND, and DSECC in the early stage of network data acquisition (between 0 and 150 times of data acquisition). However, the network energy is almost depleted when the number of times of data acquisition is 453 for the PSND algorithm, and the network still has some energy to maintain longer data acquisition for the UCBNL and DSECC algorithm. Compared with the PSND algorithm, the UCBNL algorithm can obtain longer network operation times with higher efficiency of data acquisition because the UCBNL can decrease the energy consumption of nodes during the phase of deployment by presetting the expected node numbers of each layer network and the node moving time is decreased during cluster recovery. Meanwhile, the uneven cluster deployment method can also change relay nodes by cooperating with the neighboring cluster to balance the energy consumption of the network.

#### 5.2.2. Network Coverage Rate

**Figure 9:**
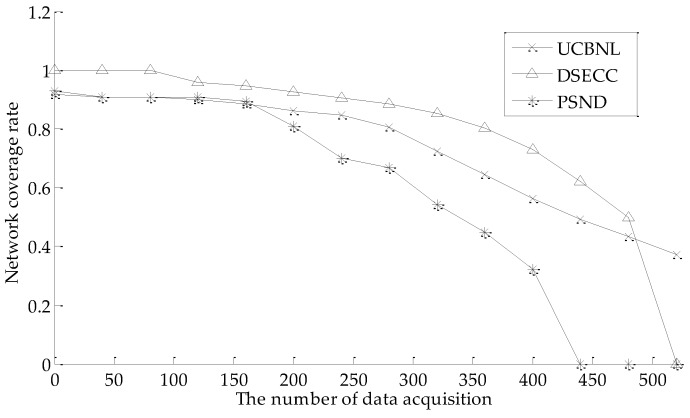
Comparison of network coverage rate during data acquisition.

Figure 9 shows that the network coverage rate and network connectivity rate varies with the number of data acquisition on UCBNL, PSND, and DSECC when the number of events is 400 and the number of nodes is 60. As shown in Figure 9, during the early phase of network operation (approximately 150 times of data acquisition previously), the UCBNL and PSND algorithms maintain similar network coverage rate and their value are high. Then, the DSECC achieves a higher network coverage rate. In addition, the network coverage rate of the PSND algorithm decreases more rapidly when the network encounters a certain phase (between 150 and 300 times of data acquisition). The network coverage rate of UCBNL decreases relatively more smoothly. The reason for this is that PSND only focuses on improving the network coverage rate, leading to excessive blind movement of nodes at the initial deployment. Although its value is high by deploying more nodes to surround all events in the early phase of network operation, the DSECC does not apply the change of network topology because of the influence of underwater flow in the later period of network operation.

#### 5.2.3. Network Connectivity Rate

**Figure 10:**
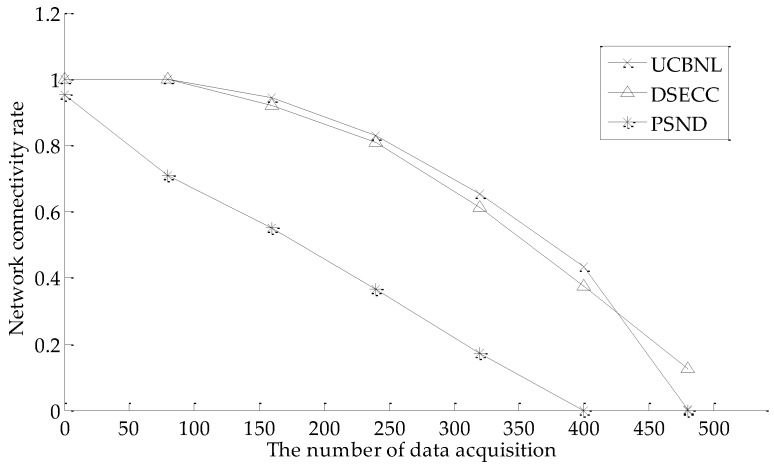
Comparison of network connectivity rate during data acquisition.

As shown in Figure 10, the UCBNL and DSECC can achieve full network connectivity in the early phase of network operation. Moreover, the UCBNL still maintains a high network connectivity rate in successive periods of longer network operation, and the network connectivity rate decreases slowly. The network connectivity rate of PSND is always smaller than that of the USBL algorithm because the UCBNL executes a heterogeneous processing of communication radii during network layering and node clustering, and the strategy of cluster recovering helps to improve the network connectivity rate. However, the PSND algorithm only ensures a high network connectivity rate by deploying more nodes to surround the events during early network operation. The PSND algorithm ignores situations wherein nodes die prematurely and cannot be recovered. Meanwhile, the DSECC can utilize the AUVs to collect data when the network approaches death.

#### 5.2.4. Network Lifetime

**Figure 11:**
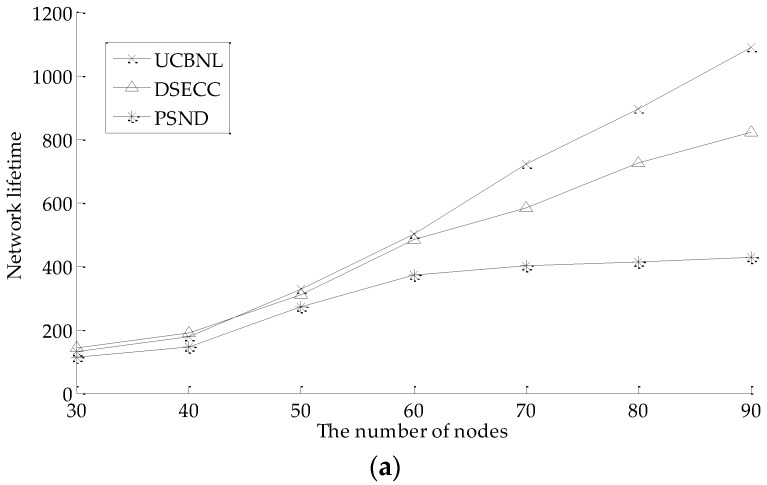

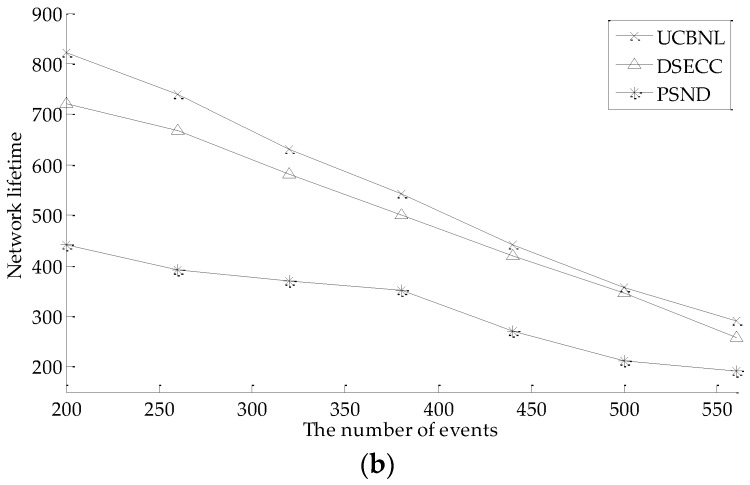
Comparison of network lifetime: (**a**) Comparison of network lifetime when the number of nodes varies; (**b**) Comparison of network connectivity rate when the number of events varies.

Figure 11a shows the comparison of the network lifetime variation with the number of nodes on UCBNL, PSND, and DSECC, where the number of events is 400 in the simulation scenario. As shown in Figure 11a, when the number of nodes is less than 45, the UCBNL, PSND, and DSECC can run close to the network lifetime. However, as the number of nodes increases, the performance of the UCBNL is superior to that of the PSND. Figure 11b shows the comparison of the network lifetime variation with the number of events on the UCBNL, PSND, and DSECC, where the number of nodes is 60 in the simulation scenario. As shown in Figure 11b, the network lifetime of the UCBNL and PSND trends downward with the growth of the number of events. We observe that the limited nodes are difficult to recharge and that blind movement will consume more energy. Evidently, the results show that using PSND results in redundant node movement. Therefore, the UCBNL will utilize more energy to perform data acquisition by decreasing the energy of node movement and prolonging the network lifetime. In addition, the DSECC cannot adopt the dynamic changes of network topology, thus accelerating network decline.

#### 5.2.5. Network Reliability

**Figure 12:**
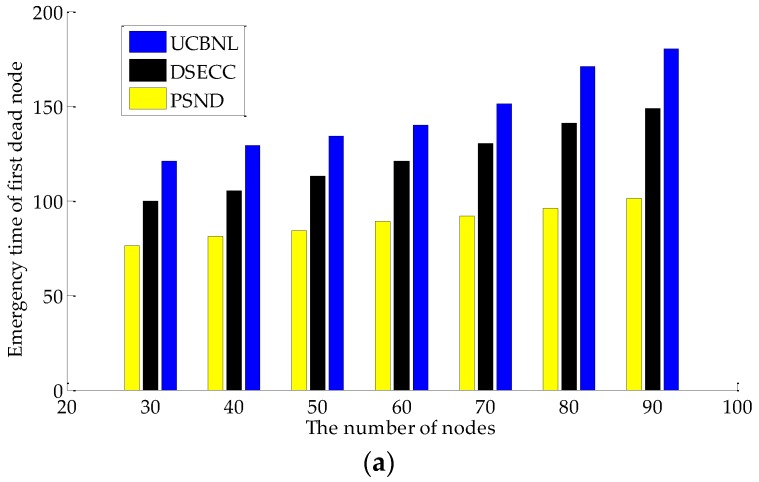

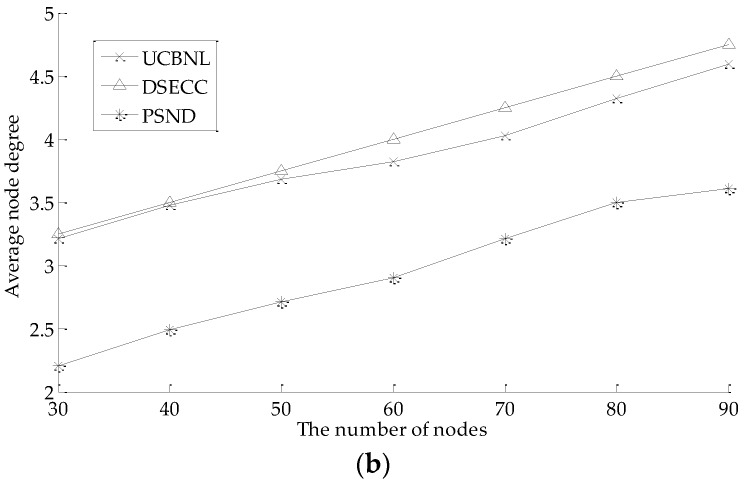
Comparison of network reliability: (**a**) Comparison of emergency time of the first dead node when the number of nodes varies; (**b**) Comparison of average node degree when the number of nodes varies.

Figure 12 illustrates the network reliability based on different indicators. Figure 12a shows the comparison of the emergency time of the first dead node variation with the number of nodes on UCBNL, PSND, and DSECC. Figure 12b shows the comparison of the average node degree variation with the number of nodes on UCBNL, PSND, and DSECC, where the number of events is 400 in the simulation scenario. These figures show that, if the number of nodes is the same in PSND, the two indicators (emergency time of first dead node and average node degree as described in Section 3.2.4) of the UCBNL is larger than that of the other algorithm. Several reasons can explain this result. First, the UCBNL is based on the analysis of Section 4, where different depths of nodes possess different network loads. The priori calculation of the expected number of nodes in each layer network can dramatically decrease the blind movement of nodes on initial deployment. Moreover, the priori calculation provides a more stable coverage density on each network for subsequent network operation to reduce the movement distance of nodes in the recovery period. Second, the process of the heterogeneous communication radius on the uneven cluster phase can replace the node that needs recovery in a timely fashion. Furthermore, flexible change network routing balances the loads of overall network data acquisition. By contrast, the PSND only operates effectively in the initial deployment by driving most nodes to cover surrounding events, with excessive energy consumption of node movement and redundancy node coverage in the initial deployment, thereby resulting in poor late network performance and network reliability.

## 6. Conclusions and Future Work

In this study, we first proposed the UCBNL to address the deployment issue of UWSNs for event coverage. This deployment of a layered mechanism avoids the large network energy consumption brought by the communication of the same layer nodes and simplifies the network model. The deployment of a layered mechanism also optimizes the network lifetime, network connectivity rate, and coverage rate. Compared with PSND and DSECC, the simulation results show that the UCBNL can maintain high network coverage and connectivity rates for a longer period of time, reduce network energy consumption, and decrease the slope of network recession, thereby prolonging network lifetime.

Several factors behind receiving distorted information will be considered in the future to account for real-life situations. These factors include underwater current, volcanic eruption, and harbor and shore activities. Such factors must be considered during node deployment in UWSNs. Meanwhile, obstacles will also be added to increase the practical applicability of the algorithm.

## Figures and Tables

**Table 1:** Simulation parameters setting.

Parameter	Value
Initial energy of node *E*_init_	1000 J
Energy threshold of node *E*_th_	20 J
Coverage threshold *C*_th_	0.1
Energy consumption on unit moving distance *m_u_*	1 J/m
Power threshold *P*_0_	0.05 W
Transmission delay *T*_p_	0.2 s
Energy spreading factor *k*	2
Carrier frequency *f*	24 kHz
Sense radius of node *Rs*	40 m

## References

[1] Garcia M., Sendra S., Atenas M., Loo J., Mauri J.L., Ortiz J.H. (2011). Underwater wireless ad-hoc networks: A survey. Book Mobile Ad Hoc Networks: Current Status and Future Trends.

[2] Bhambri H., Swaroop A. Underwater sensor network: Architectures, challenges and applications. Proceedings of the IEEE International Conference on Computing for Sustainable Global Development.

[3] Prasan U.D., Murugappan S. (2012). Underwater Sensor Networks: Architecture, Research Challenges and Potential Applications. Int. J. Eng. Res. Appl..

[4] Gkikopouli A., Nikolakopoulos G., Manesis S. A survey on underwater wireless sensor networks and applications. Proceedings of the IEEE Conference on Control and Automation (MED).

[5] Varughese A., Seetharamiah P. (2014). Design of a Node for an Underwater Sensor Network. Int. J. Comput. Appl..

[6] Hawbani A., Wang X.F., Husaini N. (2014). Grid Coverage Algorithm & Analyzing for wireless sensor networks. Netw. Protoc. Algorithms.

[7] Pinto D., Viana S.S., Nacif J.A. HydroNode: A low cost, energy efficient, multi purpose node for underwater sensor networks. Proceedings of the Conference on Local Computer Networks (LCN).

[8] Vilela J., Kashino Z., Ly R. (2016). A Dynamic Approach to Sensor Network Deployment for Mobile-Target Detection in Unstructured, Expanding Search Areas. IEEE Sens. J..

[9] Virginia P., Luigi A. (2011). Deployment of Distributed Applications in Wireless Sensor Networks. Sensors.

[10] Temel S., Unaldi N., Kaynak O. (2014). On deployment of wireless sensors on 3-D terrains to maximize sensing coverage by utilizing cat swarm optimization with wavelet transform. IEEE Trans. Syst. Man Cybern. Syst..

[11] Venkateswaran A., Sarangan V. (2008). A Mobility-Prediction-Based Relay Deployment Framework for Conserving Power in MANETs. IEEE Trans. Mob. Comput..

[12] Li H., Yang B., Chen C. Connectivity of Aeronautical Ad hoc Networks. Proceedings of the IEEE Conference on GLOBECOM Workshops.

[13] Nguyen T.T., Dao T.K., Horng M.F., Shieh C.S. (2016). An Energy-based Cluster Head Selection Algorithm to Support Long-lifetime in Wireless Sensor Networks. J. Netw. Intell..

[14] Dao T.T., Pan T.T., Nguyen T.T., Chu S.C. (2015). A Compact Articial Bee Colony Optimization for Topology Control Scheme in Wireless Sensor Networks. J. Inf. Hiding Multimed. Signal Process..

[15] Ali T., Jung L.T., Faye I. (2014). End-to-End Delay and Energy Efficient Routing Protocol for Underwater Wireless Sensor Networks. Wirel. Pers. Commun..

[16] Pompili D., Melodia T., Akyildiz I.F. Deployment analysis in underwater acoustic wireless sensor networks. Proceedings of the 1st ACM international workshop on underwater networks.

[17] Xia N. (2012). Fish Swarm Inspired Underwater Sensor Deployment. Acta Autom. Sin..

[18] Du H., Xia N., Jiang J. (2014). Particle swarm inspired underwater sensor self-deployment. Sensors.

[19] Peng J., Liu J., Wu F. (2015). Node Non-Uniform Deployment Based on Clustering Algorithm for Underwater Sensor Networks. Sensors.

[20] Bharamagoudra M.R., Manvi S.K.S. (2016). Deployment Scheme for Enhancing Coverage and Connectivity in Underwater Acoustic Sensor Networks. Wirel. Pers. Commun..

[21] Huang C.J., Wang Y.W., Lin C.F. (2011). A self-healing clustering algorithm for underwater sensor networks. Clust. Comput..

[22] Cai S.B., Zhang G.Z., Liu S.L. (2014). Weighted Localization for Underwater Sensor Networks. Lect. Notes Electr. Eng..

[23] Cheng W., Teymorian A.Y., Ma L. Underwater Localization in Sparse 3D Acoustic Sensor Networks. Proceedings of the 27th Conference on Computer Communications, INFOCOM 2008.

[24] Han G., Zhang C., Shu L. (2013). A survey on deployment algorithms in underwater acoustic sensor networks. Int. J. Distrib. Sens. Netw..

[25] Liu L. (2011). A deployment algorithm for underwater sensor networks in ocean environment. J. Circ. Syst. Comput..

[26] Alam S.M., Haas Z.J. Coverage and connectivity in three-dimensional networks. Proceedings of the 12th Annual International Conference on Mobile Computing and Networking.

[27] Alam S.M.N., Haas Z.J. (2015). Coverage and connectivity in three-dimensional networks with random node deployment. Ad Hoc Netw..

[28] Akkaya K., Newell A. (2009). Self-deployment of sensors for maximized coverage in underwater acoustic sensor networks. Comput. Commun..

[29] Senel F., Akkaya K., Erol-Kantarci M., Yilmaz T. (2014). Self-deployment of mobile underwater acoustic sensor networks for maximized coverage and guaranteed connectivity. Ad Hoc Netw..

[30] Wu J., Wang Y., Liu L. (2013). A Voronoi-Based Depth-Adjustment Scheme for Underwater Wireless Sensor Networks. Int. J. Smart Sens. Intell. Syst..

[31] Partan J., Kurose J., Levine B.N. (2007). A survey of practical issues in underwater networks. ACM Sigmobile Mob. Comput. Commun. Rev..

[32] Ahmed S., Javaid N., Khan F.A. (2015). Co-UWSN: Cooperative Energy Efficient Protocol for Underwater WSNs. Int. J. Distrib. Sens. Netw..

[33] Zhou Z., Peng Z., Cui J.H. (2010). Scalable Localization with Mobility Prediction for Underwater Sensor Networks. IEEE Trans. Mob. Comput..

[34] Bahi J., Haddad M., Hakem M. (2014). Efficient distributed lifetime optimization algorithm for sensor networks. Ad Hoc Netw..

[35] Wahid A., Lee S., Kim D. (2014). A reliable and energy-efficient routing protocol for underwater wireless sensor networks. Int. J. Commun. Syst..

[36] Saxena S., Mishra S., Singh M. (2013). Clustering Based on Node Density in Heterogeneous Under-Water Sensor Network. Int. J. Inf. Technol. Comput. Sci..

